# Dental caries in Mexican schoolchildren: A comparison of 1988–1989 and 1998–2001 surveys

**DOI:** 10.4317/medoral.18008

**Published:** 2012-05-01

**Authors:** Maria E. Irigoyen, Adriana Mejía-González, Marco A. Zepeda-Zepeda, Armando Betancourt-Linares, Miguel Á. Lezana-Fernández, Carlos H. Álvarez-Lucas

**Affiliations:** 1 Health Care Department/DCBS/Autonomous Metropolitan University−Xochimilco, México D.F. México; 2 National Center of Epidemiological Surveillance and Disease Control. Ministry of Health, México D.F. México; 3 Oral Health Office, National Center of Epidemiological Surveillance and Disease Control, Ministry of Health, México D.F. México; 4 General Direction, National Center of Epidemiological Surveillance and Disease Control, Ministry of Health, México D.F; 5 Preventive Programs, National Center of Epidemiological Surveillance and Desease Control. Ministry of Health, México D.F. México

## Abstract

Objectives: To compare two surveys across seven states for the prevalence of dental caries among Mexican schoolchildren. Study Design: Analysis of two cross-sectional surveys: Schoolchildren from 6 to 10 years of age were examined in the 1988–1989 survey and 6- to 10-year-old and 12-year-old students were included in the 1998–2001 survey. The baseline data of seven states were available for analysis. Representative probability samples were conducted statewide in both surveys. The World Health Organization (WHO) method was used to obtain the dental caries index (dmft, DMFT). At present, additional and more recent epidemiological data representative statewide in Mexico are unavailable. Results: The participants were 9798 schoolchildren in the 1988–1989 survey and 16882 schoolchildren in the 1998–2001 survey. The prevalence of caries in children ages 6 to 10 years was 86,6% in the first survey and 65,5% in the second survey, showing a 24,4% reduction. The primary teeth index in the first survey was dmft = 3,86 (IC95% 3,68 4,04) and in permanent teeth, it was DMFT = 1,03 (IC95% 0,95 1,11). In the second survey, the comparable values were dmft = 2,36 (IC95% 2,20 2,52) and DMFT = 0,35 (IC95% 0,29 0,40), corresponding to a reduction of 38,89% and 66,02% in the primary and permanent dentition, respectively. Treatment needs remain high: In the second survey, as 92,75% of the index DMFT was conformed as decayed teeth. Conclusion: Overall, we detected a downward trend in the dental caries indices, particularly in the permanent dentition. The increase in the availability of fluoride likely contributed to the observed decline in dental caries.

** Key words:**Schoolchildren, dental caries, treatment needs, salt fluoridation, Mexico.

## Introduction

Diseases of the oral cavity produce deterioration in quality of life, causing discomfort, pain, and functional limitations. These diseases also have an impact on overall health. Among Mexican children and adolescents, studies have detected an association between quality of life and caries experience (1). The treatment of oral disease is costly. It is difficult for health systems to meet the population’s treatment needs, particularly in developing countries.

Trends in dental caries have shown different patterns around the world. Several developed countries have experience a steady reduction in dental caries; however, trends in low- and middle-income counties are unclear. For the period 1970−2004, a study of three developing regions (Sub-Saharan Africa, the Middle East and North Africa, Latin America and the Caribbean) found mixed trends in the 5- to 6-year-old children but a reduction in dental caries in 11- to 13-year-olds. The lowest caries indices were found in Africa and the highest in Latin America (2).

According to the data from the World Health Organization (WHO) in 2002, the global burden of oral diseases in years lost to disability was 7372. (3) In Mexico in 2007, the Public Health Services Ministry added more than 10 million dental visits to this figure the services provided by private dentists must be added. Currently, there is one dentist for every 8911 persons, and the Ministry of Health has only 3772 dental facilities in the country. These resources are insufficient to cope with dental caries treatment needs in Mexico.

The main element used for caries prevention is fluoride; this agent has been added to several products such as dentifrice`s, rinses, gels, water, and salt (4,5). In Mexico, fluoridated dentifrice`s are widely used. Salt fluoridation programs have been implemented for more than half a century in Europe and more recently in Latin America (6,7). In 1993, Mexico established a National Salt Fluoridation Program (NSFP).

Dental caries surveys are good instruments to identify trends in dental caries. In 1988–1989, a dental caries survey was carried out including nine states of Mexico and the capital city. About 10 years later, a second survey (1998–2001) was performed that included a representative sample from each state in Mexico. Changes in dental caries indices in Mexico have been reported using the information of only three states and Mexico City; however, the rest of the states with baseline data have not been studied (8,9). These partial results showed a wide variation in caries reductions in the states studied, ranging from a reduction of 40% to 89% (8).

The comparison of the 1988–89 and 1999–2001 Mexican surveys, considering the seven states not studied so far, might provide useful information on the trend of dental caries in Mexico and offer pertinent data for the evaluation of preventive strategies and dental services. However, the evaluation of these surveys is pending; currently, more recent epidemiological data representative statewide are not available in Mexico. Likewise, the analysis of this information could be useful in designing public health policies based on scientific evidence (10). Additionally, the results could provide useful information for countries planning caries prevention strategies or intending to make changes in existing preventive programs.

The purpose of this study is to compare the results of prevalence and severity of dental caries in Mexican schoolchildren from seven states obtained in the 1988–1989 and 1998–2001 surveys to identify trends of this disease.

## Material and Methods 

Study population 

Through the Office of Prevention and Oral Disease Control, the Ministry of Health organized a baseline dental caries survey in 1988–1989 before the implementation of the National Salt Fluoridation Program. Representative samples were obtained from 11 states out of the 32 Mexican states forming the geopolitical division of the country. Partial results have been published (8,9). Analysis was pending in the states of Baja California Sur (BCS), Colima (Col.), Chiapas (Chis.), Guerrero (Gro.), Hidalgo (Hgo.), Morelos (Mor.), and Yucatan (Yuc.). The results of these seven states are analyzed in the present study. These states are located in the north (BCS and Col.), center (Mor. and Hgo.), and south (Gro., Chis. and Yuc.), covering approximately 15% of the Mexican population and representing more than 16 million people. Approximately 10 years after the baseline survey, a second survey (1999-2001) was completed.

The Pan American Health Organization (PAHO) supported both surveys, and a PAHO expert group instructed the examiners in dental caries evaluation. WHO criteria for dental caries assessment was used in both surveys. Inspection of the oral cavity was performed with natural light using a plane mirror and an explorer No. 5. The caries indices dmft (primary teeth decayed, missing, or filled) and DMFT (permanent teeth decayed, missing, or filled) were obtained. The protocol of both surveys established a prerequisite that the examiners show intra-examiner reliability of 95% and inter-examiner of 90%, which was satisfied. The survey protocols were approved by the Ministry of Health Committees, and ethical aspects were considered. During the surveys, children found in need of immediate oral care were referred to corresponding health services.

For the 1988–1989 survey, the sample size was calculated for each participating state. The sample was designed considering the information available from local studies in the region. In the second survey (1998–2001), to estimate sample size, data available from the first survey were used. The calculation of sample size was completed by the Division of Epidemiology 

of the Ministry of Health. More information can be found on the National Epidemiological Surveillance Center of the Ministry of Health website.

Data analysis 

To facilitate the comparison between surveys, the pre-valence estimates and averages for the seven states were standardized by age, using population distribution data from the XII General Census of Population and Housing 2000 as the standard. The calculations of the estimators of totals, averages, and proportions were done, taking into account the complex survey design of the surveys. The statistical analysis was performed with Stata/SE10, using the survey option (Stata Corporation, College Station, TX, USA).

## Results 

There were 9798 children participating in the first survey (1988–1989), and all were residents in the states analyzed in this study. The number of participants in these same states was 16882 in the second survey (1998–2001). ( Table 1) shows the distribution of schoolchildren by age and state.

**Table 1:**
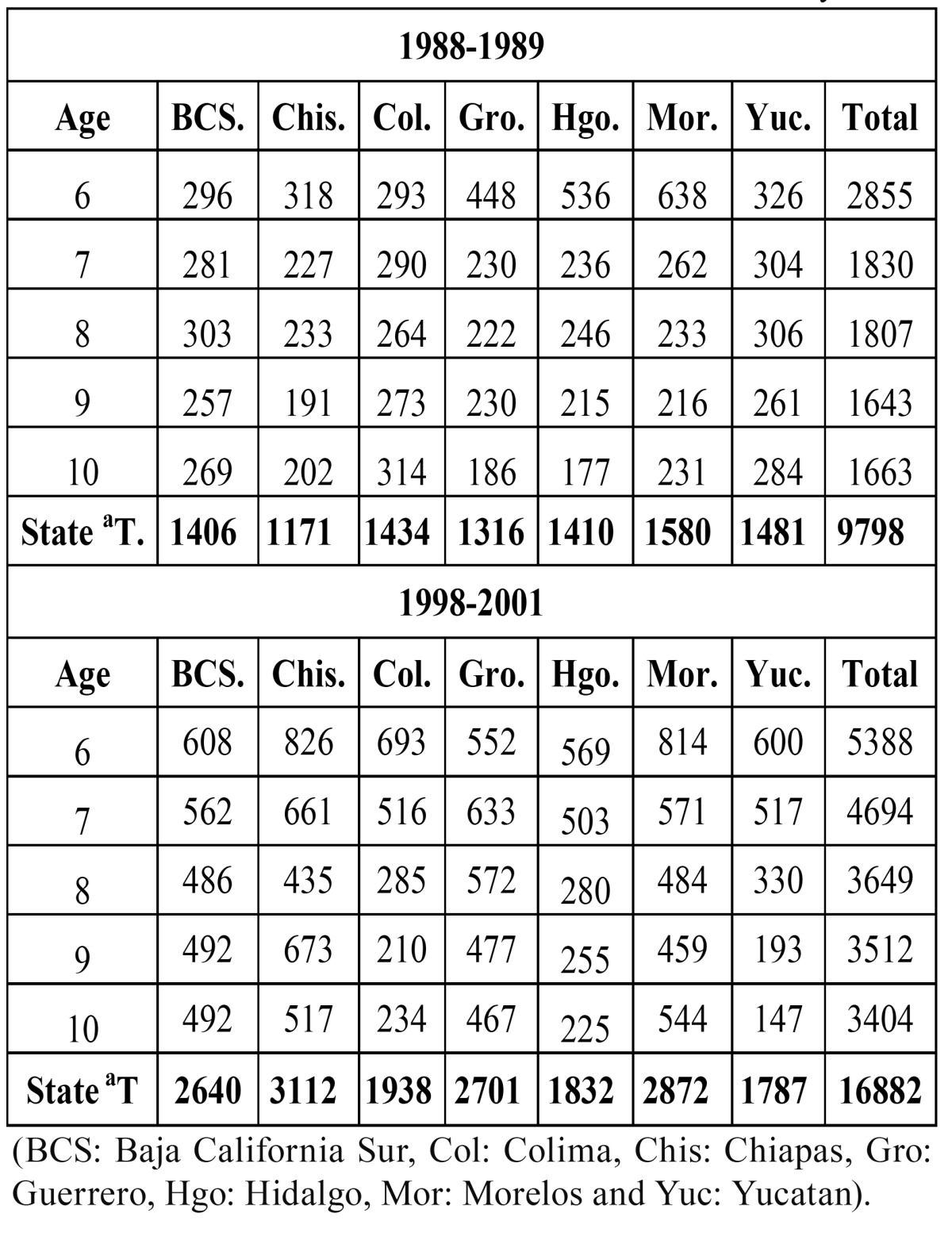
Number of participating children by age in seven states in Mexico in the 1988-1989 and 1998-2001 surveys.

Prevalence of caries

 Table 2 presents the prevalence of dental caries (dmft + DMFT> 0) by age and state in 1988–1989 and 1998–2001. In the first survey, the highest prevalence was detected in Baja California Sur and Chiapas. Data from the second survey showed that the highest prevalence was found in a midwest state, Morelos (80,3%). The state of Yucatan had the lowest prevalence (34,2%). Considering the 1988–1989 results, the caries’ prevalence was 86,6%, while in 1998–2001 it was 65,5%—a reduction in the caries’ prevalence of 24,4% in 6- to 10-year-old children.

**Table 2:**
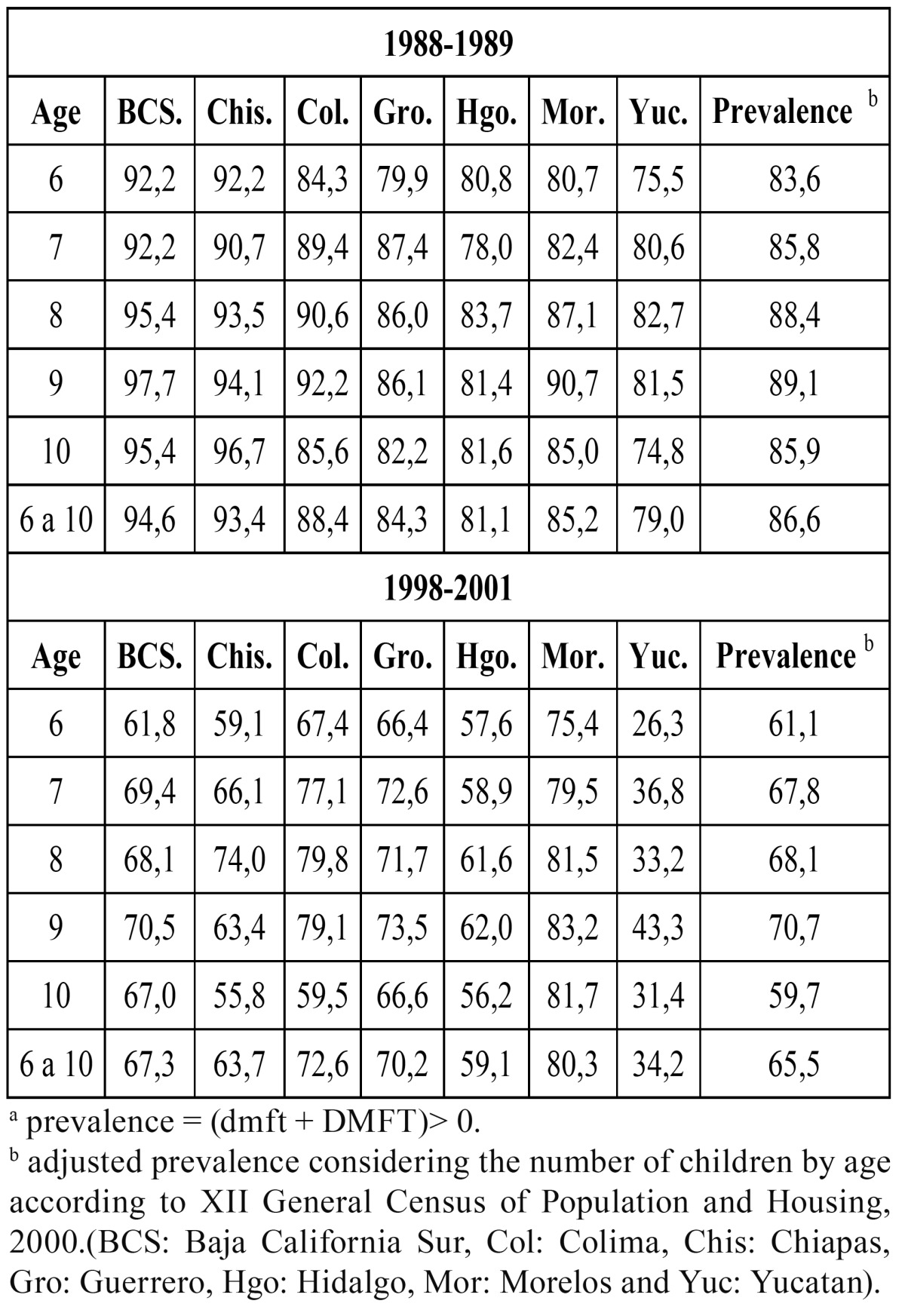
Prevalence of dental caries by age in seven states in Mexico in the 1988-1989 and 1998-2001 surveys a.

Dental caries in the primary teeth

In the 1988–1989 survey, dental caries indices were high. The groups most affected were in Baja California Sur, Colima, and Chiapas (dmft> 4), followed by Guerrero, Hidalgo, and Morelos (3 <dmft <4); the lowest state was Yucatan (dmft 2,88) ( Table 3). In the first survey, in all states, about 90% of the index was composed of decayed teeth; the filled and extracted components had only a small contribution ( Table 3). In the State of Chiapas, the percentage of extracted teeth corresponded to 8,5% of the index, while filled teeth accounted for only 0,9%.

**Table 3:**
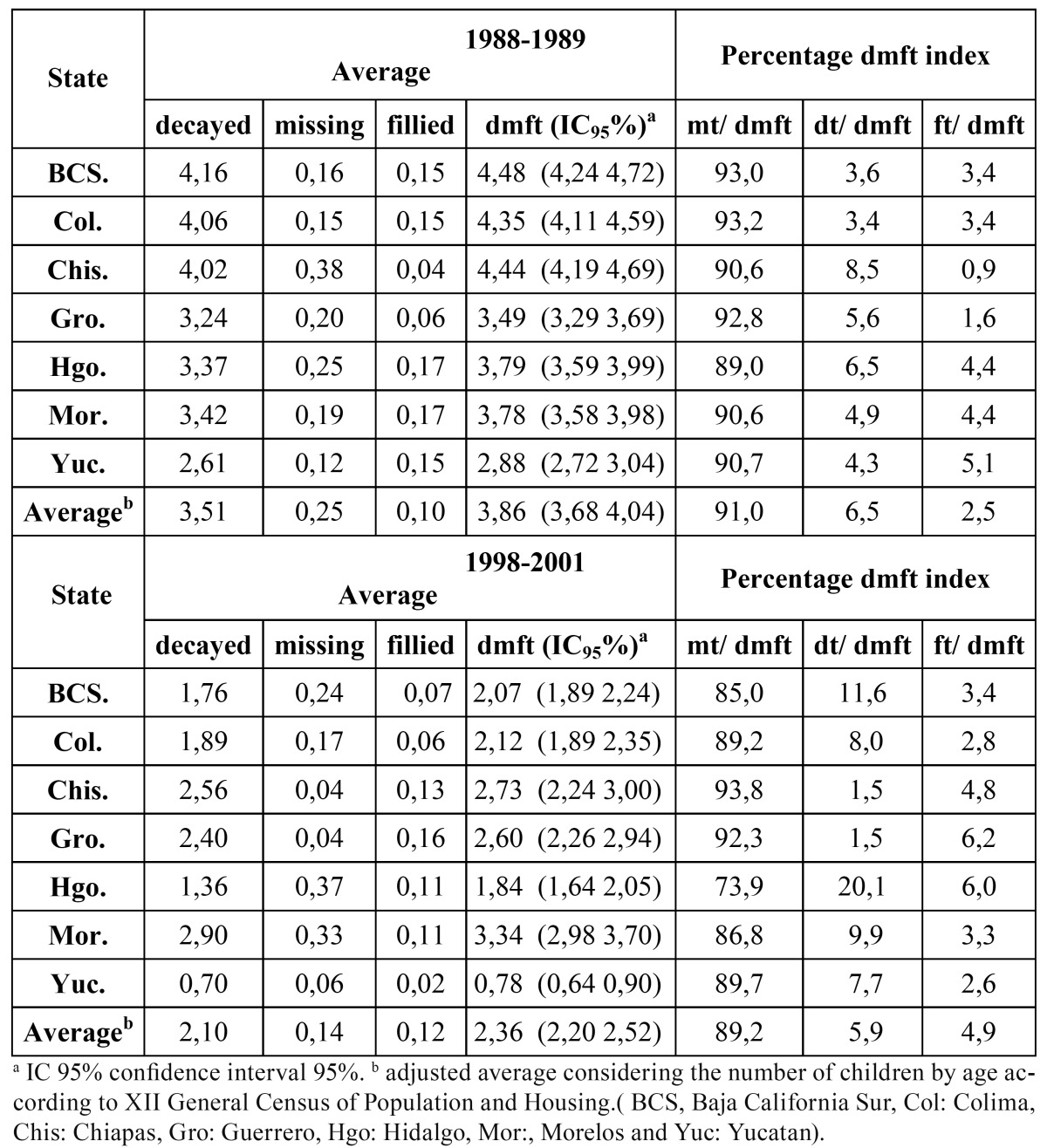
Dental caries Indices in primary teeth (dmft) and its components in 6- to 10- years-old schoolchildren in seven states in Mexico in the 1988-89 and the 1998-2001 surveys.

**Figure 1:**
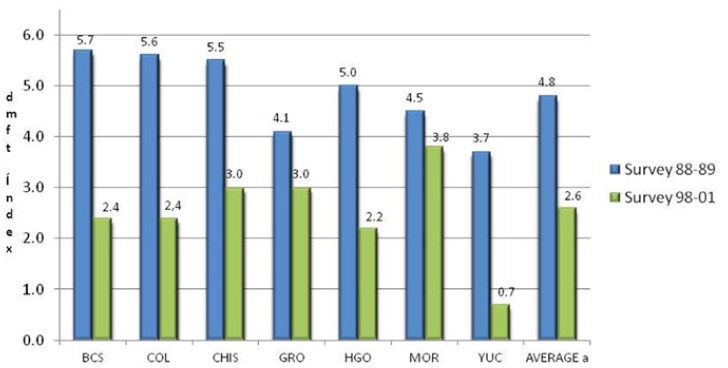
Dental caries indices in primary teeth (dmft) in 6-year-old schoolchildren in seven states of Mexico in the 1988−1989 survey and the 1998−2001 survey. AVERAGE a: Weighted average, age adjusted using as standard the XII General Population and Housing Census 2000. BCS: Baja California Sur, Col: Colima, Chis: Chiapas, Gro: Guerrero, Hgo: Hidalgo, Mor: Morelos and Yuc: Yucatan.

In the second survey, all states had a lower dmft index than in the first survey ( Table 3). In the 6- to 10-year-old children, the overall reduction in the dmft index was 38,86%. The largest reduction was observed in Yucatan (72,92%), followed by Baja California Sur (53,79%), Colima (51,26 %), Hidalgo (51,45%), Chiapas (38,51%), and Guerrero (25,50%). The smallest reduction occurred in Morelos at 11,64%. (Fig. 1) shows the caries index in primary dentition in 6-year-old children by surveys. The overall adjusted caries reduction in the 6-year-olds was 44,53%.

In addition, in the 1998–2001 survey, as in the previous survey, the component that most contributed to index dmft was decayed teeth, which accounted for approximately 90% of the index in four states (Colima, Chiapas, Guerrero, and Yucatan) ( Table 3). The component of extracted teeth was second in importance in most states studied, with the exception of Chiapas and Guerrero. In Hidalgo, more than 20% of the index was extracted teeth. In general, there were few filled teeth, and this was the component with the lowest participation in the index. In all states, this component was less than 7% of the dmft index. The increase of the filled component involvement in the dmft was only 2,4 percentage points between 1988–1989 and 1998–2001.

Dental caries in permanent teeth 

The caries indices in permanent teeth in schoolchildren ages 6 to 10 in the first and second surveys are presented in ( Table 4). In the first survey, the states of Baja California Sur and Colima showed DMFT indices greater than 2, Guerrero and Hidalgo about 1, and Morelos, Chiapas, and Yucatan less than 1. In the second survey, six of the seven states had low indices (DMFT <0,5); however, in Morelos, it was greater than 1. The overall adjusted average was 

**Table 4:**
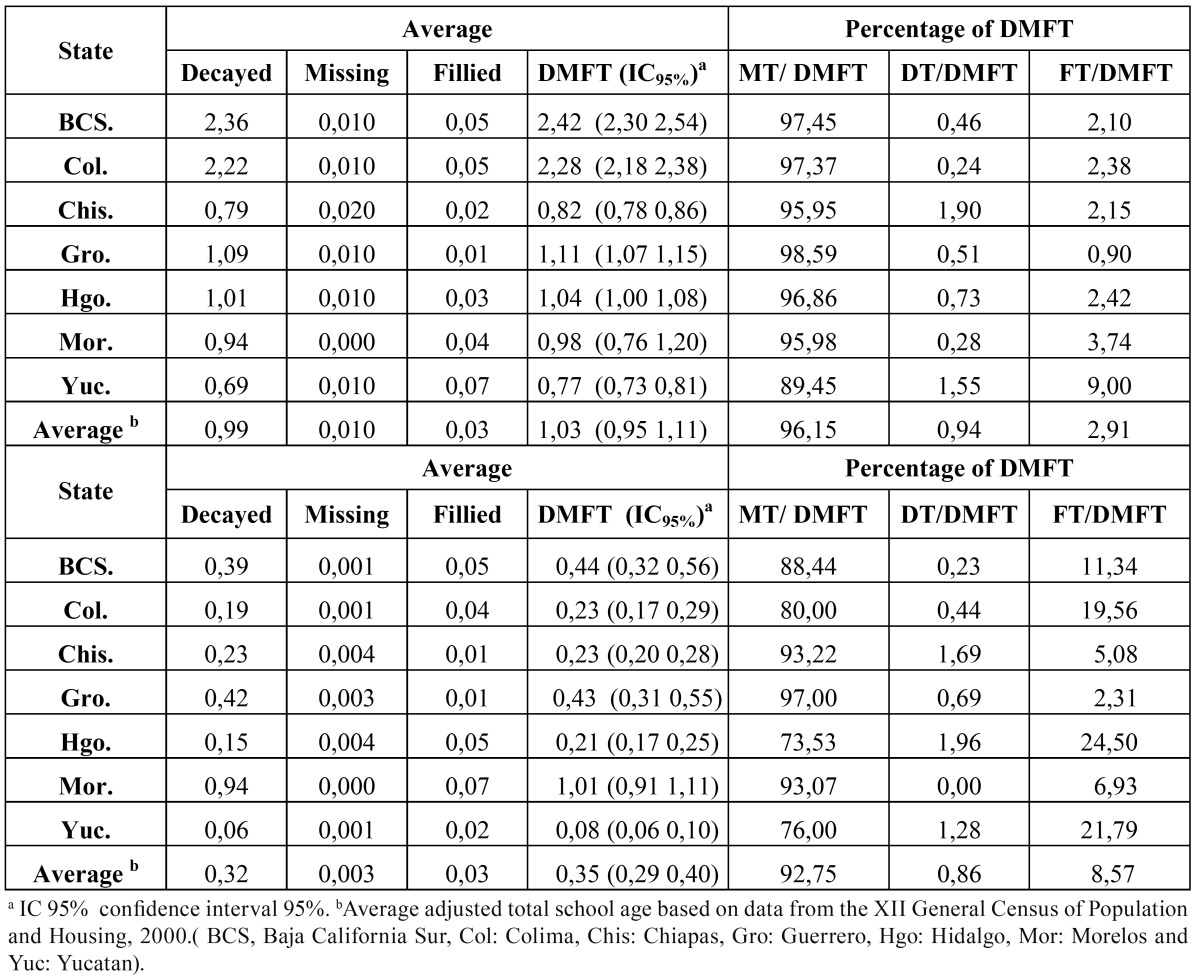
Dental caries indices in permanent teeth (DMFT) and its components in 6 to 10 years-old schoolchildren in seven states in Mexico in the 1988-89 and the 1998-2001 survey.

**Figure 2:**
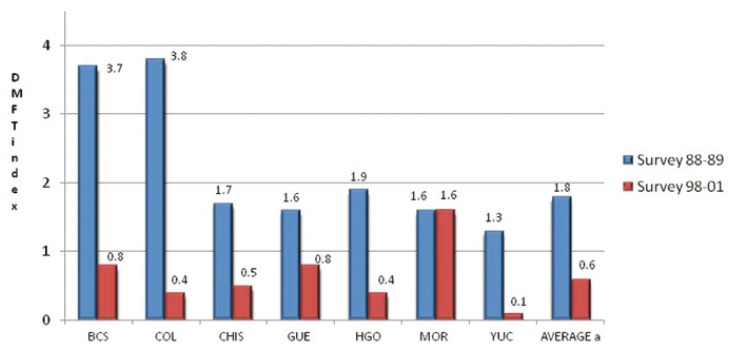
Dental caries indices in permanent teeth (DMFT) in 10-year-old schoolchildren in seven states of Mexico in the 1988−1989 survey and the 1998−2001 survey. AVERAGE a: Weighted average, age adjusted using as standard the XII General Population and Housing Census 2000. BCS: Baja California Sur, Col: Colima, Chis: Chiapas, Gro: Guerrero, Hgo: Hidalgo, Mor: Morelos and Yuc: Yucatan.

DMFT = 0,35. The reduction in caries DMFT index in the group of 6- to 10-year-olds was more than 80%, in Colima (89,91%), Yucatan (89,61%), and Baja California Sur (81,82%), followed by Hidalgo (79,81%), Chiapas (70,73%), and Guerrero (61,26%). Contrary to these trends, a rise was detected in Morelos (3,06%). The overall percent reduction was 66,02%. (Fig. 2) presents the DMFT index in 10-year-old schoolchildren by state in the 1988–1989 and 

1998–2001 surveys. Lower indices were observed in the second survey in six states; the only exception was Morelos, where no reduction was detected. The overall mean reduction in the DMFT was 61,4% in 10-year-old children.

The second survey included 12-year-old students in the results of the DMFT index. In this group, the indices were Baja California Sur (1,54), Chiapas (1,36), and Guerrero (1,33), followed by Colima (0,76) and Hidalgo (0,74). The highest DMFT was observed in Morelos (3,25); Yucatan (0,52) had the lowest index. The mean DMFT at age 12 was 1,33 in the population studied in the seven states.

The treatment needs in the permanent dentition in the study period remained high ( Table 4). The filled component accounted for less than 10% of the index in three states (Chiapas, Guerrero, and Morelos), and the rest of the states were between 10% and 25%. The increase in filled component between the two surveys was 5,66 percentage points.

## Discussion

In the 1988–1989 survey, the means caries prevalence in the population studied was high (86,60%), while in 1998–2001 the prevalence was 65,50%. A comparison of these surveys showed an average caries index reduction of 25%. However, according to data from the second survey, certain regions of the country still had high caries prevalence.

Dental caries in primary dentition

In 1988–1989, the average index of caries at 6 years was high (dmft = 3,86) and treatment needs were also high. The percentage of filled teeth was low, especially in Chiapas, where filled teeth were identified less frequently than missing teeth. In the second survey, there was a decrease in the percentage of extracted teeth; however, filled teeth were still a small part of the caries index.

In the second survey, the overall caries dmft index was 2,36. In Hidalgo, a high proportion of extracted teeth was found (20,1%). This was likely because when parents took their children to the dental office, they already had advanced dental destruction in primary teeth, which reduced the possibility of conservative treatment; extraction`s were carried out to cope with the problem.

The 1998–2001 survey showed that among the states considered, there was a wide variation in the caries indices and in the caries reduction detected. This variation emphasizes the multi factorial nature of dental caries, as individual susceptibility, diet, oral hygiene, access to health services, and sociocultural aspects, among others, have important roles in development of the caries process (5,11). These aspects varied among the states surveyed.

In general, the reductions in caries indices were lower for primary teeth than for permanent ones. In the U.S. population, information from national surveys of 1988–1994 compared with 1999–2004 showed an increase in the rate of decay in primary teeth, and this increase was also identified in the Mexican-American group of children (12,13). Attention should be given to dental caries in primary dentition—not only because of the discomfort it causes children but also because this is an important risk factor for development of caries in permanent teeth (14,15).

Dental caries in permanent teeth

The results of the 1998–2001 survey showed low caries indices in the permanent dentition in several states; however, in others, high caries indices were still present. The comparisons of the surveys indicated that in the 10-year-old children, there was an annual decrease of 6,6% in the DMFT index. This decrease is lower than that observed in other countries with salt fluoridation programs such as Costa Rica, where there was an annual reduction of 8,3%—much lower than 15,2% reduction in Jamaican 12-year-old children (16,17). It is worth mentioning that the salt fluoridation program in Jamaica not only added fluoride to table salt but also required this salt in restaurants and various food products (17). The caries reduction observed in Mexico is close to that found in early studies on the impact of the salt fluoridation program in Colombia (18). In a period of eight years, 10-year-old Colombian children showed a 58,5% caries reduction. Over a period of 10 years in Mexico, the reduction was 65,59%.

In the present study, the State of Morelos was the only place with no significant reductions in the caries index; indeed, the results revealed a slight increase in the caries index in permanent teeth in some age groups. Of the seven states studied, Morelos is the closest in proximity to Mexico City. It is possible that the population of Morelos has moved toward a diet similar to that of children in Mexico City, changing their traditional diet—tortilla, beans, and some vegetables—to a sucrose-rich, high-caloric density diet containing large quantities of fermentable carbohydrates, accompanied by an increase in the frequency of food consumption (19). These features of the modern diet have been shown to be risk factors for dental caries (20).

During the study period, the low caries index found among children in the State of Yucatan and their tendency toward a marked caries reduction (89,6%) are findings for which causes have yet to be elucidated. The Indian heritage of Yucatan children might be a factor that influences the phenomenon. interestingly, similar results were found with the salt fluoridation program in Guatemala, a country that borders the states of southeastern Mexico and the people share their indigenous origins.

The caries index at 12 years of age is used by WHO as an indicator for international comparisons of trends of this disease. In 2000, the WHO goal for the DMFT index was three or fewer teeth affected. The results of the second survey in Mexico showed that six of the seven states studied had reached this goal. The average DMFT was 1,33 for 12-year-old students. This index is close to that observed in the United States in the National Survey 1999–2004 of caries (DMFT = 1,4). The results of Mexico compared with Costa Rica (DMFT = 2,5) were lower and close to Jamaica (DMFT = 1,1). Further, 12-year-old Mexican children reached the goal for the year 2020 of the WHO European Regional Office (DMFT = 1,5); however, the 6-year-old children studied were far from the WHO goal of at least 80% caries free (21).

More studies are needed to elucidate the role of preventive measurements in the dental caries decline observed in Mexico. The surveys analyzed did not gather sufficient information regarding the children’s use of dental caries preventive measurements (e.g., use of fluoridated dentifrice, fluoridated salt consumption, professional applied fluoride, etc). However, we can speculate that most of the children used fluoridated dentifrice`s because the majority of the dentifrice`s in Mexico contained fluoride in their formula, and these products are widely sold. In addition, the consumption of fluoridated salt is basically guaranteed, because it is largely the only type of salt available in the states surveyed due to the national regulations. The salt fluoridation program covers more than 80 million Mexicans.

Treatment needs

The need for dental treatment experienced a slight decrease in all states in the study period but remained high; in three states, the filled component did not exceed 10% of the DMFT. Particularly in states with high percentage of population living in poverty (Guerrero and Chiapas), the majority of children had never received restorative treatment, which is expressed in a low filled component that did not reach 5% of the DMFT. Other Latin American countries in the 1990s experienced similar situations—Belize, Bolivia, Ecuador, Honduras, Nicaragua, Panama, and Paraguay—as the percentage of filled component comprised less than 10% of the DMFT. In contrast, in the United States (1999−2002), in 6- to 11-year-old children, 73,8% of the DMFT index was filled teeth (22).

Study limitations

Among the limitations of this study are those related to the long period required to gather the data (1998–2001), which was the result of different constraints on economic resources and the dental personnel available in the states. However, once the survey was initiated, the field phase was completed in months. In addition, it is possible that dental caries diagnosis criteria varied between the first and second surveys, which set limitations in the comparison of results. However, an effort was made to avoid this problem, and both surveys used the same WHO criteria for diagnosis of caries, and the examiners were trained by some of the same PAHO experts.

One of the strengths of the study is the size of sample, which involved numerous localities and examined a large number of children in different parts of the states. This resulted in estimators with good levels of precision.

The results showed a dental caries prevalence reduction of approximately 25% in the population studied. It is difficult to establish the accurate contribution of the different preventive factors that have led the trend of the decline. In addition to fluoridated salt, the population has made extensive use of fluoride tooth pastes. Further, the health services have specific preventive programs in elementary schools and low-income areas. Surveillance of the caries prevention programs should be carried out to evaluate the benefits of the programs, detect groups with greater needs, and identify the communities with higher risk of dental fluorosis.
